# Osteonecrosis of the external auditory canal in two patients on denosumab therapy

**DOI:** 10.1093/jbmrpl/ziaf134

**Published:** 2025-08-18

**Authors:** Holly Wang, Matthew Yii, Bing Mei Teh, Erosha Premaratne, Cherie Chiang

**Affiliations:** Department of Endocrinology, Austin Health, Melbourne, 3084, Australia; Department of ENT, Austin Health, Melbourne, 3084, Australia; Department of ENT, Austin Health, Melbourne, 3084, Australia; Department of Otolaryngology, Head and Neck Surgery, Monash Health, Faculty of Medicine, Nursing and Health Sciences, Monash University, Clayton, 3168, VIC, Australia; Department of Endocrinology, Austin Health, Melbourne, 3084, Australia; Department of Medicine, University of Melbourne, Melbourne, 3050, Australia; Department of Endocrinology, Austin Health, Melbourne, 3084, Australia; Department of Medicine, University of Melbourne, Melbourne, 3050, Australia

**Keywords:** osteonecrosis of the external auditory canal, osteonecrosis of the jaw, antiresorptive agent, bone turnover marker, osteoporosis

## Abstract

Medication-related osteonecrosis of the jaw (MRONJ) is a rare but well-recognized complication of treatment with antiresorptive agents. Medication-related osteonecrosis of the external auditory canal (MROEAC), on the other hand, is even rarer and mostly reported during bisphosphonate exposure. Its pathophysiology is thought to involve complex multifactorial processes, including inhibition of bone remodeling, altered angiogenesis, infection, and inflammation. Due to the poor recognition of MROEAC, ear canal health is not routinely discussed with patients, and there is no requirement for auditory canal assessment prior to antiresorptive therapy. We present 2 cases of denosumab-associated MROEAC: a 72-yr-old woman with osteoporosis treated with denosumab for 5 yr (Case A), and an 84-yr-old man with osteoporosis treated with denosumab for 6 yr (Case B), both of whom presented with unilateral ear itch and were subsequently diagnosed with MROEAC. Both patients were managed conservatively with topical corticosteroid, antimicrobial, antifungal ointments, and cessation of denosumab with subsequent elevation of bone turnover markers. In Case A, a course of teriparatide immediately after denosumab cessation did not improve bone erosion. Teriparatide was ceased due to patient’s concern with renal calculi, high bone resorption markers, and vertebral fracture. A trial of risedronate was commenced with reduction of bone markers and epithelization of the auditory canal. In Case B, risedronate was commenced with reduction of bone markers and early healing of the auditory canal at 4 mo. Osteonecrosis of sites other than the jaw should be considered a potential rare complication and balanced against the bone protection benefits of long-term antiresorptive therapy. The optimal treatment strategy for MROAEC remains uncertain. Currently, there is no recommendation for routine auditory canal assessment prior to or during antiresorptive treatment. However, sequencing off established denosumab use with follow-on treatment to mitigate potential rapid bone loss needs to be considered.

## Graphical Abstract

**Graphical Abstract:**
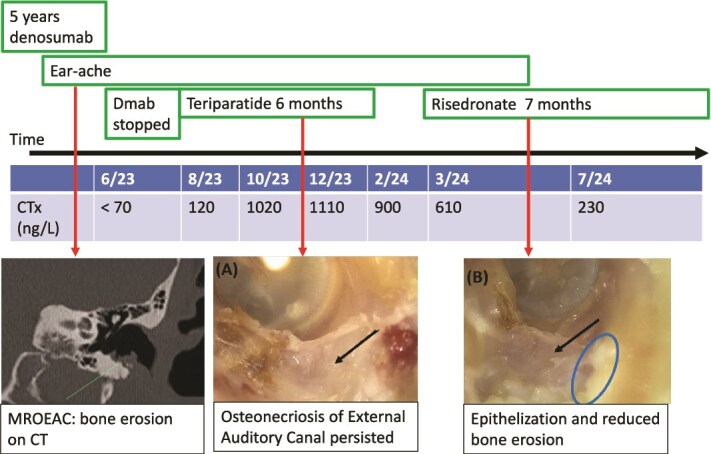

## Introduction

Bisphosphonates and denosumab are medications used commonly in the management of osteoporosis, bone metastases, and other bone disorders. It is widely recognized that these medications are associated with an increased risk of medication-related osteonecrosis of the jaw (MRONJ).^1^ Routine dental screening is recommended prior to commencing treatment with antiresorptive agents, and there is guidance around the management of patients at risk of MRONJ.^2^ However, a similar but rarer complication is medication-related osteonecrosis of the external auditory canal (MROEAC),^3^ which to date has been described in over 30 case reports using terminology, such as “oral bisphosphonates-associated ear canal osteonecrosis,”^4^ the majority of which were associated with bisphosphonates.^3^^,^^5–9^ This lesser-known complication is likely under-reported, and may present with otalgia, otorrhoea, hearing loss, or facial nerve dysfunction; occasionally it is asymptomatic and detected incidentally.^3^^,^^5^^,^^6^ We describe two cases of unprovoked MROEAC associated with denosumab use to highlight the complexity of follow-on treatment in view of the high bone loss post denosumab cessation.

## Case description

### Case A

A 72-yr-old female presented with a 5-yr history of worsening left ear itch, without otalgia, otorrhoea, or traumatic procedure to the canal. She commenced 60 mg denosumab injections administered every 6 mo in 2018 (5 yr duration) for her osteoporosis and previous fragility fractures of radius and metatarsal. Other comorbidities included stable monoclonal gammopathy of unknown significance (MGUS) (IgM kappa 15 g/L) with mild suppression of IgG (5.43, reference range: 6.1-16.2 g/L), bilateral symmetrical high frequency sensorineural hearing loss without the need for hearing aid, chronic obstructive pulmonary disease, gastro-esophageal reflux controlled on 20 mg daily of pantoprazole, and renal calculi 10 yr ago. There was no exposure to systemic glucocorticoid therapy.

**Figure 1:**
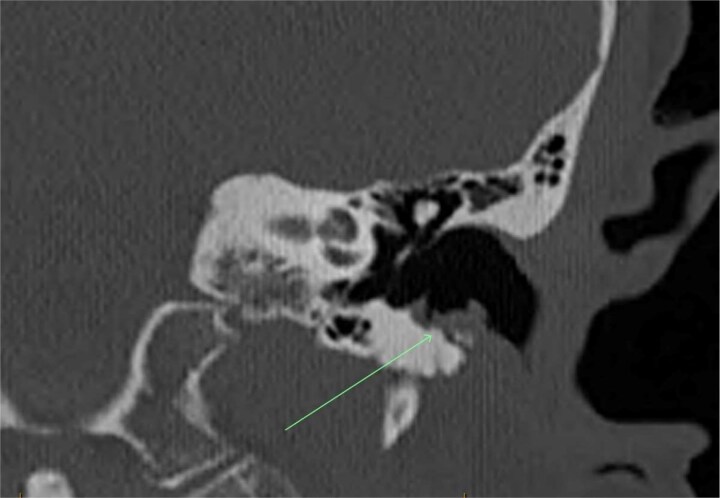
Case A—coronal CT scan of left ear canal, arrow demonstrating area of inferior canal erosion with overlying lobulated soft tissue.

A review by an Ear, Nose, and Throat (ENT) surgeon via otoscopy noted erosion and exposed bone of the floor of the left external auditory canal in May 2023. Computed tomography imaging confirmed the bony erosion and soft tissue changes that supported the diagnosis (Figure 1). The erosion was managed conservatively with topical Kenacomb Otic ointment (triamcinolone acetonide 0.1% + neomycin 0.25% + gramicidin 0.025% + nystatin 100 000 units/g), instilled during each ENT review. A clinical diagnosis of MROEAC was made, thought to be secondary to denosumab use.

**Figure 2:**
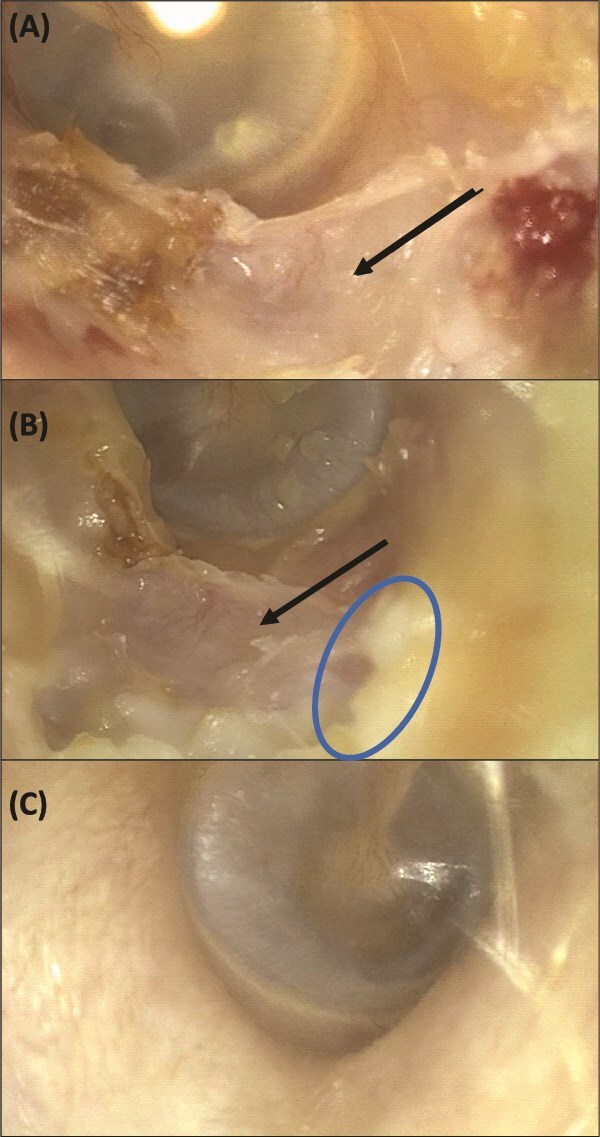
Otoscope images of ear canal of Case A. (A) Left external auditory canal 1 mo post risedronate treatment: normal tympanic membrane located medially with skin defect and exposed external auditory canal bone inferiorly (black arrow). (B) Left external auditory canal 7 mo post risedronate treatment: early epithelization (blue circle) over the reduced bone defect (black arrow). (C) Normal right external auditory canal.

In light of the diagnosis of osteonecrosis of external auditory canal, denosumab was ceased with last dose in June 2023, and daily 20 mcg subcutaneous teriparatide injection was commenced in the same month. She developed transient hypercalcemia with corrected calcium of 2.75 mmol/L (reference range*:* 2.10-2.60 mmol/L) and was not taking calcium supplementation at that time. Additionally, she developed multiple symptomatic bilateral calcium oxalate renal calculi requiring stents in November 2023. Teriparatide was ceased in December 2023 due to patient’s concerns with hypercalcemia and renal calculi. The patient was reluctant to continue teriparatide at the current dose, or at a reduced dose frequency. Investigations at the time of teriparatide cessation, which was 6 mo after the last dose of denosumab, noted low urinary calcium excretion of 2.0 mmol/d (reference range: 2.5-7.5 mmol/d), normal urinary oxalate excretion, elevated C-terminal telopeptide of type 1 collagen (CTx) of 1110 ng/L (reference range: 100-1000 ng/L) and elevated procollagen type 1 amino-terminal peptide (P1NP) of 132 μg/L (reference range: 15-115 μg/L). Her DXA scan (Hologic) at that time demonstrated T-scores of −1.6 at the left FN, −2.2 at the lumbar spine, and −0.9 at the distal radius. Compared to 2018 when DXA (Lunar) was performed on a different analyzer, the baseline T scores were −1.6 and −1.9, respectively, in the FN and LS. No fractures were seen on X-rays of the pelvis, bilateral femurs, and thoracolumbar spine while the area of exposed bone in the left auditory canal was unchanged after cessation of denosumab and teriparatide (Figure 2A). The right auditory canal was normal (Figure 2C).

The patient was informed that the interpretation of bone loss trend was limited due to images produced by different DXA manufacturers. However, due to concerns with rebound high bone turnover and bone loss with the sequence of denosumab to teriparatide treatment, balanced with concerns for worsening of the osteonecrosis of the auditory canal, she commenced risedronate in February 2024 with regular ENT follow-up for ear toilet and application of Kenacomb Otic ointment. Risedronate was well tolerated without gastro-esophageal reflux symptoms, blood tests performed after 6 mo of commencement showed decline in bone turnover markers (Table 1) with early healing and epithelization on repeat otoscopic examinations (Figure 2B). At the last review in June 2025 (17 mo after risedronate), there is ongoing improvement of skin regeneration and only a small area of exposed bone remains inferiorly (otoscopy image not available).

**Table 1:** Change in bone turnover markers for Case A and B.

	Timepoint 1	Timepoint 2	Timepoint 3	Reference range
**Case A**	Dec 2023: After 6 mo of teriparatide and 6 mo post cessation of denosumab	Feb 2024: 1 mo of risedronate	July 2024: 6 mo of risedronate	
**CTx (ng/L)**	1110	610	230	100-1000
**P1NP (mcg/L)**	132	91	30	15-115
**Case B**	November 2024: 6 mo post last DMAB dose	January 2025: 8 mo post last DMAB dose	March 2025: 2 mo of risedronate	
**CTx (ng/L)**	110	460	160	100-750
**P1NP (mcg/L)**	13	34	50	15-115

### Case B

**Figure 3:**
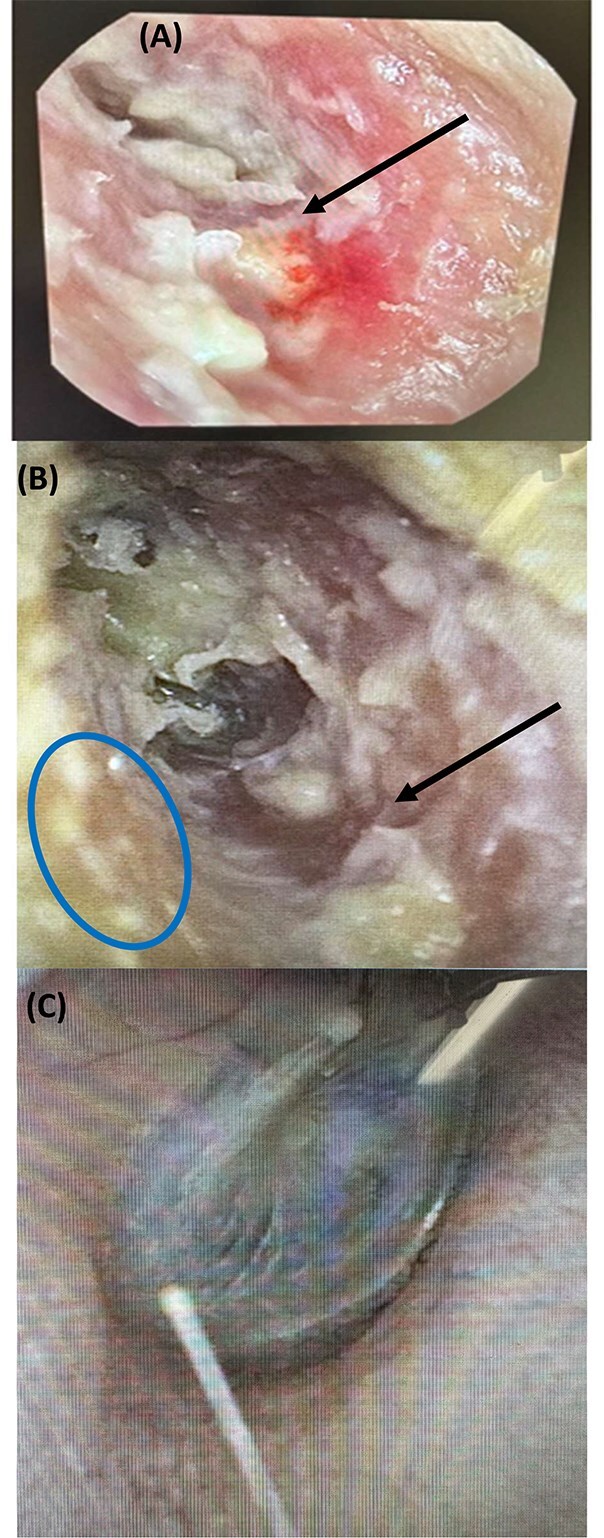
Otoscope images of ear canal of case B. (A) Left external auditory canal while on denosumab: tympanic membrane located medially obscured by debris, skin defect and bone exposure (black arrow). (B) Left external auditory canal 6 mo post risedronate: early epithelization (blue circle) over the reduced bone defect (black arrow). (C) Normal right external auditory canal.

An 84-yr-old man was referred to an ENT surgeon with a 2-yr history of left ear discomfort and itch. He commenced 60 mg denosumab injections administered every 6 mo in 2018 (6-yr duration) for osteoporosis without fragility fractures. At baseline in 2018, bone density T-score was −2.7 at the LS, and this improved in 2021 (same Lunar analyzer) to −2.0 on denosumab treatment. There was no preceding trauma to the ear canal, no cotton bud use, and no exposure to systemic glucocorticoids. His comorbidities included osteoarthritis of knees, dyslipidemia, impaired fasting glucose, ischemic heart disease, vitamin B12-deficiency, inguinal and femoral hernias, prostate hypertrophy, and rectal adenocarcinoma cured after anterior resection. Otoscopic examination demonstrated a large amount of debris, with a small area of bone exposure in the inferior section of the left external auditory canal (Figure 3A). Given the clinical diagnosis of left-sided MROEAC, he was treated with topical ointment and referred to our endocrinology service to revisit his denosumab therapy.

The decision was made to cease denosumab (last dose May 2024), with close monitoring of bone turnover markers and instillation of Kenacomb Otic (triamcinolone acetonide 0.1% + neomycin 0.25% + gramicidin 0.025% + nystatin 100 000 units/g) at monthly ENT visit. Risedronate was commenced January 2024 (8 mo after the last denosumab dose) when CTx rose above 50% of mid normative range (Table 1). Teriparatide therapy was discussed, but not pursued given patient’s age and preferences. He commenced on risedronate 35 mg weekly with reduction of CTx, resolution of earache, and reduction of exposed bone with early epithelization after 4 mo (Figure 3B). The right external auditory canal remained normal (Figure 3C).

## Discussion

Medication-related osteonecrosis of the jaw is a well-described complication of antiresorptive agents. Its incidence is approximately 1%-15% in the oncology patient population, and 0.001%-0.01% in the osteoporosis population and is more likely to occur with cumulative dose of exposure.^1^ Bisphosphonates have been reported to cause osteonecrosis in unexpected sites including the thumb and calvarial bone.^10^^,^^11^ Case reports of bisphosphonate-related osteonecrosis affecting the auditory canal date back to 2007,^7–9^ and it was first reported in a patient using denosumab in 2021.^5^ Its working definition is bone of the ear canal that is exposed for greater than 8 wk in patients using high-risk medication (bisphosphonates, denosumab, bevacizumab, temsirolimus, and sunitinib), in the absence of previous radiotherapy in the temporal area, exclusion of metastases, and cholesteatoma; it is a clinical diagnosis whereby radiographic and histopathological findings are supportive but not required for diagnosis.^6^^,^^8^

The underlying pathophysiology for MRONJ and MROEAC is believed to be similar and involves multiple processes including inhibition of bone remodeling, altered angiogenesis, infection and inflammation, and altered immune function.^1^ Infection and inflammation are thought to be key components in the process of osteonecrosis, although it is unclear whether necrosis follows infection, or vice versa. In areas of dental infection, bacterial microfilms have been observed to be a site of osteoclast activity and bone resorption, and most MRONJ cases are provoked by a dental procedure. In the limited series of reported MROEAC, trauma to the ear canal with a cotton bud was noted in only one case,^4^ although not all case reports specified whether potential ear canal trauma had occurred. Since trauma is not always implicated, MROEAC might be preceded by infection and inflammation of the external auditory canal, also known as otitis externa. There are distinct risk factors for otitis externa, such as narrow ear canal, eczema of the ear canal, hearing-aid users, swimmers, and humid environment. The microbiological profile is also different in the chronic otitis externa compared to dental infection, often with mixed fungal and bacterial infections (eg, *Pseudomonas aeruginosa*, *Staphylococcus aureus*).^12^ The oral cavity on the other hand is a reservoir of bacterial pathogens (oral microbiome), and dental infection is therefore polymicrobial with a mixture of anaerobes.^13^ Furthermore, bisphosphonates may inhibit proliferation of oral keratinocytes and thus disrupt oral mucosal integrity;^1^^,^^14^ it is unclear whether a similar process may take place within the ear canal. In the case series by Froelich et al., i.v. bisphosphonates used in oncological conditions was associated with MROEAC.^7^ High cumulative dose of antiresorptive use in the oncology population is also a known risk factor for MRONJ, leading to the ASCO Clinical Practice Guideline.^15^ Antiangiogenic action of bisphosphonates may also contribute, and similarly, antiangiogenic agents sunitinib and bevacizumab have been reported to cause MRONJ and MROEAC; however, evidence to support this process is mixed, and denosumab is not known to have any antiangiogenic effects.

Onset of symptoms varies widely. Presenting symptoms include otorrhoea, otalgia, hearing loss, facial nerve palsy; some cases are asymptomatic.^6^ Duration of symptoms varied from weeks to years prior to diagnosis (Table 2). In approximately 50% of cases, osteonecrosis was present in bilateral auditory canals.^6^ Risk factors are similar to those for MRONJ and include glucocorticoids, local irradiation, smoking, as well as local trauma, such as cotton bud use.^5–9^ Unlike MRONJ, systemic conditions, such as diabetes mellitus and rheumatoid arthritis, have not been reported as a key feature.^1^^,^^14^ Case A has stable IgM kappa MGUS with suppression of IgG that increases the risk of infection. Monoclonal gammopathy is known to reduce osteoblast activity and increase fracture risk.^16^ MGUS may have contributed to MROEAC independent of denosumab use in this case.

**Table 2:** Summary of MROEAC cases associated with denosumab use.

Author	Patient age and sex	Duration of denosumab therapy	Trauma	Site	Symptoms (duration)	Treatment	Outcome
**Wang et al.**	72F	5 yr	No	Left	Otalgia (5 yr) Unrelated bilateral sensorineural hearing loss	Denosumab cessation, topical ointment, teriparatide for 6 mo, risedronate for 17 mo	Almost healed after 17 mo of risedronate with epithelization and small area of exposed bone remaining inferiorly
**Wang et al.**	84M	6 yr	No	Left	Otalgia (2 yr)	Denosumab cessation, topical ointment, risedronate for 4 mo	Healing on repeat otoscopy after 4 mo of risedronate
**Takeda et al.^5^**	81F	1.5 yr	No	Bilateral	Otalgia, otorrhoea, mixed conductive and sensorineural hearing loss (1 yr)	Denosumab cessation, topical ointment, left radical mastoidectomy	Left resolved post-surgical debridement. Right progressed slowly with conservative management
**Kumar et al.^6^**	79F	2 yr	No	Bilateral	Nil	Denosumab cessation, topical ointment	Stable at 12 mo
**Kumar et al.^6^**	64F	3 yr (preceded by 5 yr Risedronate)	No	Right	Otalgia	Denosumab cessation, topical ointment	Stable at 12 mo
**Peřina et al.^17^**	68F	3 yr	Not reported	Right and (+ right MRONJ)	Otalgia, otorrhoea (4 mo), asymptomatic MRONJ	Denosumab cessation, surgical resection of mandible, surgical debridement and skin graft of right auditory canal	Resolved post-surgical debridement
**True et al.^22^**	79F	4 mo (preceded by Alendronate >10 yr)	No	Bilateral	Otalgia, bilateral conductive hearing loss (<2 mo)	Denosumab cessation, topical ointment, IV and oral antimicrobials	Completely healed at 12 mo Bilateral conductive hearing loss also resolved

Response to topical therapy was variable in the literature. While ear swabs are not routinely taken as culture results do not alter management, anti-microbial ointment is instilled to cover the bone defect and re-applied at each ENT visit to promote healing and prevent infection. Oral and i.v. antibiotics are reserved for malignant otitis externa. Some cases of MROEAC required local debridement or more extensive surgery.^6^^,^^17^ Surgical debridement, such as mastoidectomy, is necessary in cases of obvious dead bone or sequestrum, if malignancy is suspected or if progression is supported by CT or bone technetium imaging. Canalplasty to widen a narrow external auditory canal can be performed to reduce recurrent otitis externa. During otoscopy in Case A and B, initial visualization of healthy exposed bone in a normal diameter canal and clinical improvement with skin regeneration gave reassurance to continue conservative topical therapy. In published case reports of MROEAC in antiresorptive users, the need for surgical management did not seem to be predicted by the cumulative exposure to antiresorptive, presence of risk factors, or presenting symptoms.^4^^,^^5^^,^^8^^,^^9^^,^^18^^,^^19^ Due to its rarity and under-recognition by clinicians, ear canal osteonecrosis may be misdiagnosed as cholesteatoma, malignant otitis externa or temporal bone malignancy.^18^

There is a potential role for teriparatide in facilitating healing of MRONJ. Sim et al. conducted a placebo-controlled RCT in which 34 participants with a total of 47 MRONJ lesions were randomized to receive either 8 wk of teriparatide or placebo injection.^20^ The trial included patients on bisphosphonates and denosumab, with treatment indications including osteoporosis and malignancy. Teriparatide was associated with a greater rate of resolution of MRONJ lesions at 12 mo (45.4% vs 33.3%, odds ratio 0.15 vs 0.40; *p* = .013).^20^ A retrospective review by Morishita et al. in 2020 that included 29 individuals with MRONJ treated with teriparatide for a median of 14 mo (range: 0.3-26 mo) found that it was beneficial for 75.9% of patients, and led to complete resolution in 65.5%.^21^ Teriparatide use has not been reported in MROEAC. While the 6 mo of teriparatide use (truncated due to intolerance) did not result in healing in Case A, its lack of efficacy cannot be generalized to other MROEAC cases. Pending the availability of randomized controlled head-to-head studies comparing risedronate, teriparatide, and conservative antiresorptive cessation, it is difficult to know which strategy promotes the best healing.

Only 5 other cases of MROEAC associated with denosumab use have been reported,^5^^,^^6^^,^^17^^,^^22^ one case suffered concurrent MRONJ and MROEAC while on denosumab 120 mg every 3 mo for metastatic breast cancer.^17^ Denosumab was ceased in all cases with variable outcomes (Table 2), the most severe case required left radical mastoidectomy for temporal bone necrosis. None of the published cases reported subsequent changes in bone turnover markers and strategies to mitigate rebound high-bone-turnover and bone loss after denosumab cessation.

High bone turnover was noted post teriparatide use in Case A, and 8 mo after denosumab dose in Case B. Teriparatide alone is not effective in counteracting the known rebound bone loss phenomenon following denosumab cessation; furthermore, this sequence of teriparatide after denosumab has been demonstrated to cause bone loss particularly at the radius site.^23^ This poses a management dilemma if further antiresorptive agents are contraindicated due to MROEAC in light of current recommendations for bisphosphonate therapy consolidation after cessation of established denosumab use.^24^ The natural time course of MROEAC is not well described, it is unknown whether it improves with antiresorptive cessation, recurs with antiresorptive recommencement, whether teriparatide expedites healing or the optimal timing of surgical intervention when conservative topical therapies fail. Some of the cases were asymptomatic and MROEAC was noted incidentally, it is uncertain if progression will occur if antiresorptive agents were continued. In MROEAC cases where antiresorptive agents were ceased, there was a range of outcomes including rapid improvement, healing within 6 mo, and stability at 12 mo.^4–6^^,^^18^^,^^25^ In the few reported cases of MROEAC in which bisphosphonates were continued,^9^^,^^22^^,^^25^ healing or stability was also noted. In our two cases of mild non-suppurative MROEAC and rebound high-bone-turnover after cessation of denosumab, the decision was made to trial risedronate due to its lower antiresorptive potency and shorter half-life, with ongoing regular otoscopy to monitor the site of osteonecrosis. The previously elevated CTx declined to below the 50th percentile of pre-menopausal range in both cases. For Case A, the auditory canal was stable throughout the course of denosumab cessation and 6 mo of teriparatide. Early epithelisation over the bone defect and decline in CTx were noted after 7 mo of risedronate. Due to Case A’s concern over the risk of vertebral fracture after denosumab cessation, it was important to balance the loss of bone density if teriparatide was ceased without antiresorptive consolidation therapy,^23^ and the potential risk of MROEAC progression with antiresorptive recommencement. Although there is no randomized controlled trial involving risedronate in MROEAC, we observed ongoing auditory canal improvement and bone turnover markers suppression in our two cases after up to 17 mo of risedronate therapy.

Finally, it is prudent to note that while MROEAC is rare, otitis externa is common and affects 10% of the population at some stage during their lifetime.^26^ Media coverage of MRONJ and atypical femoral fracture previously induced unfounded fear of the generally safe and effective osteoporosis medications and led to decline in usage.^27^ While ear-itch or discomfort is common, the education and communication between physicians and patients regarding MROEAC need to be delivered with a clear message to emphasize the importance of osteoporosis treatment while sensibly reporting rare side effects.

In conclusion, external auditory canal osteonecrosis is a rare and probably under-recognized complication of antiresorptive agents. While a dental check is recommended before antiresorptive treatment, universal auditory canal assessment is unlikely to be cost-effective due to the expertise required. Management of established cases is usually supportive with a lack of guidelines regarding subsequent osteoporosis treatment. Clinician awareness and further studies to assess the evolution of MROEAC with antiresorptive cessation and teriparatide, or risedronate treatment are required for the management of these patients. The overall skeletal benefits of antiresorptive agents should be balanced against the rare side effect of MROEAC during patient education.

## Data Availability

Data available on request.
